# Music Self-Efficacy for Performance: An Explanatory Model Based on Social Support

**DOI:** 10.3389/fpsyg.2020.01249

**Published:** 2020-06-25

**Authors:** Francisco Javier Zarza-Alzugaray, Oscar Casanova, Gary E. McPherson, Santos Orejudo

**Affiliations:** ^1^Department of Musical, Plastic and Body Expression, University of Zaragoza, Zaragoza, Spain; ^2^Melbourne Conservatorium of Music, University of Melbourne, Melbourne, VIC, Australia; ^3^Department of Psychology and Sociology, University of Zaragoza, Zaragoza, Spain

**Keywords:** self-efficacy, social support, performance anxiety, music education, structural equation modeling

## Abstract

Personal perceptions of self-efficacy are particularly relevant in the field of music performance, which is oriented toward the outward expressions of one’s own ability through public performances. Within this context, a number of personal variables, including social support and performance anxiety, have been shown to be associated with musical success and are therefore relevant for research that seeks to understand the four sources of self-efficacy (mastery experiences, vicarious observation, verbal persuasion, physiological states) that are integral components of Bandura’s (2002) Social Learning Theory. Previous research, as well as observed differences among musicians associated with educational level (preuniversity) and gender (male/female), underpins the context of this study, which presents evidence regarding the factors that are capable of mediating perceptions of self-efficacy for musical performance. Specifically, the main objectives of this study were to more clearly understand relations between social support, public performance, musical performance anxiety, and self-efficacy using structural equation modeling and to compare these results according to gender. A battery of questionnaires was submitted to 359 preuniversity Spanish music students. Results highlight the relevance of family support for self-efficacy in public performance: directly and mediated through musical performance anxiety. The role of teachers and peers appeared to be relevant only for boys and was mediated through performance anxiety. Public performances lead to a greater degree of musical self-efficacy, but only in girls. Further research shall be required in order to improve pedagogical methods and help teachers increasingly individualize their teaching.

## Introduction and Rationale

Determining factors that influence the attainment of success has become increasingly prominent in studies within the psychopedagogical field (Zimmerman, 2000). Achieving mastery in any discipline is challenging and becomes even more complicated in the field of music, where it is necessary to acquire a series of complex skills that are then put on display during public performances and formal recitals. Many years of training and preparation are required to acquire such skills and put these into practice, a process that involves not only the development of technical dexterity, but also the ability to regulate one’s learning activity and to control emotional factors (Hallam, 2002). Personal motivation and the context in which learning takes place also exert a pivotal influence throughout this entire process (Moore et al., 2003; Creech and Hallam, 2011; Lehmann and Kristensen, 2014; Hallam et al., 2016).

The analysis of musical self-efficacy, for example, of the perception of one’s own competence to perform in front of an audience or to prepare such a performance via a learning process (Ritchie and Williamon, 2011), provides an opportunity to gain a more in-depth understanding of these personal dimensions in music students. Musical self-efficacy is significantly related to performance (McPherson and McCormick, 2006) and involves an elevated motivational component, which regulates the goals and efforts involved in learning activities, among other factors. Additionally, the theory of self-efficacy provides us with a basis to analyze the series of factors that are involved in the construct and that thereby play a significant role in musical training. Bandura (1986, 2002) proposes four sources of self-efficacy: the individual’s own direct experiences (mastery experiences), self-modeling (vicarious experience), persuasion by others (social persuasion), and emotional factors (physiological and emotional states).

To gain an understanding of relevant personal dimensions, it is important to analyze the perceptions music students hold of their own competence to perform in front of an audience or to prepare for a performance (Ritchie and Williamon, 2011). This is because personal perceptions of one’s own ability – what researchers refer to as self-efficacy – have been found to be significantly related to performance achievement (McPherson and McCormick, 2006). In addition, the theoretical constructs related to self-efficacy provide us with a basis to analyze the four defined sources that play a significant role in musical training: the individual’s own direct experiences (mastery experiences), self-modeling (vicarious experience), persuasion by others (social persuasion), and emotional factors (physiological and emotional States) (Bandura, 1986, 2002).

The aim of this study was to apply the framework of the self-efficacy model to analyze the role that social support plays within it. Social support has been shown to be a key factor in the development of musical training (Ryan et al., 2000; Moore et al., 2003; Sichivitsa, 2007; McPherson, 2009; Lehmann and Kristensen, 2014). Such support is particularly relevant because musical training typically begins at a very early age: generally around the age of 7 years and at a stage where decision-making does not directly depend on the child, but on adults. After having taken initial decisions, the individual then invests a considerable amount of time in music training, while coordinating and adjusting the time dedicated to music with other school activities, managing stage fright [musical performance anxiety (MPA)], persevering in the face of difficulties, and, ultimately, deciding whether to embark on a career within the musical profession. Throughout this process, social support emerges as a key element; nevertheless, evidence for that important role is scant (Moore et al., 2003; Lehmann and Kristensen, 2014).

In this study, we add two further relevant variables to analyze self-efficacy. The first of these is MPA, one of the most relevant variables in the context of musical performance (and in the abandonment of musical careers); as mentioned above, MPA has a direct relationship with self-efficacy. Kenny has established an explanatory framework for MPA, which involves direct personal experience, as well as individual characteristics, specified as early context experiences, helplessness, and anxiety cognitions (Kenny et al., 2004; Orejudo et al., 2017). We sought to analyze the direct relation of MPA with self-efficacy on the basis of Bandura’s theory as a source of information, without ignoring the issue that self-efficacy is dynamic: therefore, inverse relationships might also be possible, as suggested by González et al. (2018). The second variable we took into account concerns the role played by musical performances, which should be direct and therefore, as such, have an effect on musical self-efficacy. Among all types of musical performance, solo performances are the most relevant (Casanova et al., 2018). For this reason, we included the dimension of music solo performance in our study.

**FIGURE 1:**
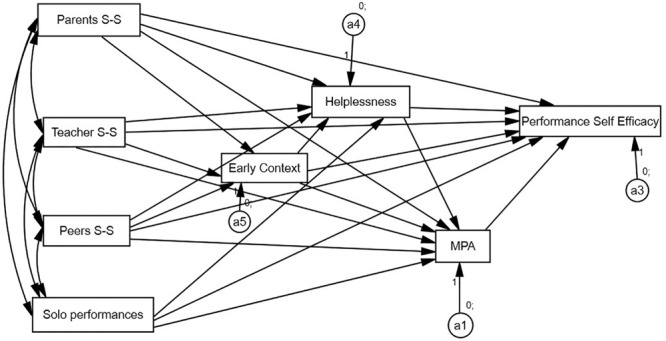
General common model.

Along with this set of variables, we propose a structural equation model (SEM) that allows us to analyze the role of several exogenous variables, mediation variables, and endogenous variables (Mulaik, 2009; Byrne, 2010). In our case (Figure 1), the endogenous variables are related with several sources of social support and with the direct experience of performance, whereas MPA – more directly related with self-efficacy, as well as with mastery experiences and with social support – acts as a mediation variable.

The SEM models are also useful to help ascertain whether the relations established therein are different among groups. The interest in our case is centered on analyzing whether the postulated relations are the same for men and for women. No previous similar types of studies have analyzed these relationships in the same manner, particularly the relationships between these variables and gender. Further evidence nevertheless points toward differences between boys and girls in this context; thus, the need to expand research in that direction is justified. For example, studies by Osborne and Kenny (2008); Thomas and Nettelbeck (2014), and Coşkun-Şentürk and Çırakoğlu (2018) point out the importance of gender as a variable that predicts MPA, as well as the importance of musical solo performances for musical self-efficacy.

### Musical Self-Efficacy

Within the extensive literature on motivation, Bandura’s (1977) provides a distinctive framework in which self-efficacy is defined as judgments that individuals make about their own personal abilities to reach a specific level of performance (Bandura, 1986). Applied to music performance, self-efficacy can be seen as a result of one’s awareness of one’s own musical abilities. That is, in the process of achieving musical success, personal characteristics or musical abilities are important, but self-efficacy emerges as an essential element that exerts a profound influence on each individual’s way of thinking, behaving, and feeling (Carbonero and Merino, 2008).

Such success must be understood as the achievement of certain goals: an ideal common to all people and an intrinsic motivation that leads to specific behaviors (Prieto, n.d.). However, being aware of these goals or the best way to achieve them is not sufficient. The perception of one’s own ability is postulated as an important mediator for successfully developing the actions that lead to the achievement of one’s personal goals (Bandura, 1977).

Obviously, competent functioning requires a systematic balance between actual capacity or effectiveness and the metacognitive processes that underpin one’s personal beliefs. These are two different but reciprocal, mutually reinforcing elements (Gálvez et al., 2005). As shown by McPherson and McCormick (2006), personal perceptions about one’s capacity to perform a musical work in public are more important predictors of musical performance success than the amount of practice (see also McCormick and McPherson, 2003). In turn, the actual achievement obtained in such an interpretation will positively or negatively affect self-efficacy beliefs. Therefore, perceptions of self-efficacy affect not only one’s choice of activities and behaviors, but also the effort invested in them, one’s thought patterns, and one’s emotional reactions (Tejada, 2005).

Specificity is a salient feature of self-efficacy, a characteristic that differentiates it conceptually and psychometrically from other neighboring constructs such as self-concept (Schunk and Pajares, 2001). Both constructs contribute to motivation (Bandura, 1987); the latter, however, demonstrates greater predictive power in relation to musical achievement (McPherson and McCormick, 2000, 2006). Self-efficacy beliefs respond to questions of the type “I can,” whereas self-concept is evaluated through questions of the type “I am” (Olaz, 2001). In other words, self-efficacy is not considered to be a global or holistic trait such as self-concept, but rather a construct of specific tasks or situations (Bandura, 1986), the dynamism and malleability of which have aroused great interest in the educational community (Berry and West, 1993). For this reason, Bandura (1986) recommends gathering self-efficacy judgment information immediately before subjects undertake a test or perform in a field of activity and using scales in which they are asked to estimate the degree to which they “can” successfully accomplish a challenging activity.

For all these reasons, it is not surprising that ever since the 1970s a significant amount of literature has been published on self-efficacy in the context of education, empirically confirming the relationship between self-efficacy and achievement (Greenberg, 1970; Svengalis, 1978; McCormick and McPherson, 2003; Nielsen, 2004; Hendricks, 2009, 2014; Zelenak, 2011; Miksza, 2015). All these studies have confirmed the influence of musical self-efficacy on achievement, yet few studies have investigated the possible factors capable of explaining the levels of self-efficacy in musicians. As music psychopedagogical research has shown, further personal, cognitive, or contextual factors need to be assessed in the accomplishment of the aforementioned achievement independently or through their mediation on an individual’s personal perceptions of their own ability.

Previous mastery experiences, vicarious observation, verbal persuasion, and physiological states are seen within Bandura’s (1977) theory as the four principal sources of self-efficacy (Bandura, 1977), and from this perspective, we can use these constructs to analyze and ascertain some of the factors involved in musical achievement. These sources of information provide an appropriate opportunity to operationalize psychological constructs such as social support and stage anxiety.

Proposing progressive goals to tackle difficulty in order to avoid experiences of failure (mastery experiences), participating as an equal in different activities (vicarious experiences), using vocabulary, adopting a positive predisposition that improves motivation (verbal persuasion), and mediating the perception of stress (physiological states) are some of these possible forms of influence of social support and MPA on subjects’ perceptions of musical self-efficacy and, ultimately, on their own musical achievement. In this sense, MPA is a significant factor in the explanation of burnout in music students (Fehm and Schmidt, 2006; Osborne, 2016).

### Social Support

Popularly used in a generic and intuitive way, the psychological construct of social support is defined as the presence or relative absence of psychological support resources from other significant people (Caplan et al., 1975). In his study with talented young people, Bloom (1985) concluded that the gifted students who had achieved mastery were not always those who displayed the most talent. Similarly, as indicated by Lehmann and Kristensen (2014), the biographies of some of the great musicians throughout history coincide in the relevance of “shadow people” in their successful musical careers. Those who achieve success in a given field are situated in and supported by a particular, ideal social context (McPherson et al., 2012; McPherson, 2016). As Bloom (1985, cited in Sosniak, 2006) reflected at the end of his project, his quest to understand exceptional children led him to discover and focus on the exceptional conditions that underpin exceptional talent.

An analysis of social support within the context of musical education requires that we take into account who are the agents of social support and what kind of support they are providing. In terms of the identity of the agents, an ample consensus in this field postulates that social support can stem from parents, teachers, and peers, although these three types of agents do not have the same relevance for academic success; nor are they equally significant as sources of different types of support (Creech and Hallam, 2011; Lehmann and Kristensen, 2014; Nogaj and Ossowski, 2015). Sichivitsa (2007) upholds those authors’ approaches by pointing out the same three sources of support as relevant for musical motivation, specifying that there can be a relationship between parent and teacher support and that the parents’ involvement in music can be a key factor in developing that support.

Apart from analyzing who are the sources/agents of support, if we want to understand their relationship with self-efficacy, we also need to analyze what functions such support provides. Different types of support can have informative, instrumental, evaluative, or emotional functions (Sęk and Brzezińska, 2008; Nogaj and Ossowski, 2015). Informative support consists in an exchange of information, guidance, and counsel that help individuals better understand their situation, taking into account their personal situations and own problems, as well as feedback concerning the effectiveness of strategies that have been used to deal with a situation. Instrumental support is constructive advice that is pertinent at the moment when trying to solve a problem. Evaluative support is the transmission of information that evaluates the capacities or strategies that are being used, thereby implying a persuasive aspect in terms of the individual’s own capacities. The fourth category, emotional support, is one of the most relevant: it implies the transmission of love, care, trust, and empathy in adverse situations. Creech (2009) provides her own theoretical model of the attitude of parents toward the musical education of their children and enumerates three types of social support: behavioral, cognitive-intellectual, and personal. Behavioral support is the type of support in which parents play a role similar to that of a teacher, but at home, for example, by helping their child with the organization of studies or homework. Intellectual or cognitive support consists in planning many different types of activities that allow the child to develop musical abilities: attending concerts, listening to recordings, or participating in extracurricular musical activities. The third type, personal support, consists in assistance provided in establishing goals and expectations, as well as encouraging the child’s comprehension and motivation related with the goals that have been attained.

Apart from these different types of social support, this article analyzes the influence of parents, teachers, and peers (friends) on musicians’ perceptions of their own self-efficacy. Via the four sources of information on self-efficacy, each of these social agents is thought to exert an influence on the achievement of music performers’ optimal state of subjective perception of their own competence, which are critical in the optimization of performance. Self-efficacy beliefs are both a cause and a consequence of success; each of the aforementioned social agents can contribute not only to the musician’s adequate perception of achievement, relating it to internal, specific, and controllable behaviors, but also to failures, turning them into experiences of controlled failure, and thus strengthening the musician’s own perceptions of self-efficacy (Rozalen, 2009).

### Music Performance Anxiety

Because of its negative influence on musical performance (Dalia, 2004; Zarza et al., 2016), performance anxiety has been regarded as one of the most important mediating influences on music performance. In recent decades, however, musical self-efficacy has been postulated as another key element in the process of achieving musical mastery (McCormick and McPherson, 2003; McPherson and McCormick, 2006). The construct of MPA points to the relevance of the emotional dimension in an individual’s success in the musical domain – a dimension likewise highlighted by other authors. As Cohen and Bodner (2019) point out, Seligman (2011) proposed a model based on the framework of positive psychology and built on four fundamental pillars for the achievement of an optimal level of performance: positive emotion, engagement, relationships, and meaning and achievement. Each of those pillars can be linked with social support and with the four sources of self-efficacy (mastery experiences, vicarious observation, verbal persuasion, physiological states) as described by Bandura (2002).

The connection with the fourth source of self-efficacy (physiological states), as well as the large body of literature on this topic within a musical context, justifies the inclusion of the MPA variable in this study. Among the different proposals or theoretical models of stage anxiety, we chose to follow Barlow’s (2000), as adapted to the musical context by Kenny et al. (2004). This model differentiates between specific contextual factors, biological factors, and general environmental factors, whereby its interaction with other individual variables is responsible for developing catastrophic interpretations of reality, to a greater or lesser extent (Zarza et al., 2016; Orejudo et al., 2018). Without pursuing it as a main objective, we analyzed the possible relationship between the two constructs of musical self-efficacy and MPA, attempting to determine if elevated perceptions of musical self-efficacy are able to mediate on the achievement of success by decreasing anxiety levels. According to Bandura (2002), it is not the intensity of emotional and/or physical reactions that matters, but rather how they are perceived and interpreted. Among others, Sinden (1999); McQuade (2009), and Stevanovic (2014) have empirically verified the explanatory capacity of musical self-efficacy regarding MPA. In this sense, the work of González et al. (2018), developed within the context of Spain, can be considered as a reference, because it applies the model of Pekrun and Linnenbrink (2012) and Zeidner (2014) in which self-efficacy predicts MPA, which, in turn, precedes the type of stimulation prior to a performance that is capable of improving performance.

The Investigating Musical Performance or IMP project (Welch et al., 2008a, b) within the framework of the Teaching and Learning Research Program or TLRP13 can be considered as a reference in pedagogical-musical research. Those studies take a sample of musicians (students and professionals) from the United Kingdom working in different musical genres (classical music, popular music, jazz music, and traditional Scottish music) and analyze which factors are capable of influencing learning, interpretation, and musical achievement. These studies, among others, highlight the need to include a series of sociodemographic, pedagogical, and family variables to achieve a more profound understanding of the psychological constructs of musical self-efficacy and social support.

In summary, despite its positive influence on motivation, achievement, and personal well-being (Bandura, 1994), self-efficacy is not regarded as the determining factor that corrects all problems, because their relationship with other personal variables or psychological constructs is what determines the equation of musical achievement. In view of all the above and because of the scant number of investigations in this regard in the Spanish context, we consider it necessary to expand our knowledge of musical self-efficacy for interpretation, whereby the main objective of this study is to analyze its relationship with other sociodemographic and pedagogical variables, as well as with social support and MPA, in order to shed light on sex differences. In this way, our research aims to clarify the types of influences that impact on the musical training undertaken by Spanish conservatory students.

## Materials and Methods

### Participants

The sample comprised 359 pupils enrolled in four preuniversity music training centers in Spain. There were 161 (44.8%) males and 198 (55.2%) females. They ranged in age from 11 to 52 years, with a mean age of 17.03 (*SD* = 5.25) years. The six academic years in Spanish music schools are grouped into three cycles (*n* = 3): 25.9% of the pupils (*n* = 93) were enrolled in the first cycle, 39.6% (*n* = 142) in the second one, and 34.5% (*n* = 124) in the third and final cycle.

### Variables and Materials

For this investigation, we used a pen-and-paper battery of questionnaires specifically designed to fulfill the purpose of this study. The battery includes the Spanish-language adaptation of the Kenny Music Performance Anxiety Inventory in its 24-item version (Cronbach α = 0.866) (Zarza et al., 2016). This scale comprises three subscales: the Early Context Subscale (three items; Cronbach α = 0.0568) evaluates the subject’s attitude toward public musical performance in relation with early music training context; the Helplessness Subscale (10 items; Cronbach α = 0.789) evaluates general aspects stemming from the general psychological vulnerability factor described in the theory of anxiety propounded by Barlow (2000) and adapted to a musical context by Kenny et al. (2004); the third subscale (11 items; Cronbach α = 0.868) measures the specific factor of anxiety associated with proximal performance concerns. The questionnaire uses a Likert-type 7-point scale: high scores indicate higher levels of anxiety, and *vice versa*.

We incorporated into the study the Spanish-language adaptation (in press) of the Self-Efficacy Music Performance Scale originally devised by Ritchie and Williamon (2011). In its final version in Spanish (Cronbach α = 0.773), the scale features 10 items designed to evaluate subjects’ opinions about their self-efficacy when confronted with the challenge of having to perform music in public. The items are measured on a 7-point Likert scale: high scores indicate high levels of self-efficacy for music performance.

We also used the Spanish adaptation (in press) of the social support scale developed by Ryan et al. (2000). This 7-point Likert scale test was designed to evaluate the degree of social support perceived by music students. In its final Spanish version, it comprises several independent subscales related to different social agents such as parents (nine items; Cronbach α = 0.849), professors (nine items; Cronbach α = 0.866), and peers (three items; Cronbach α = 0.934).

Finally, we gathered descriptive data including age and gender, as well as the number of solo public performances the subject had given in the course of the preceding academic year, by asking the question: “How many solo performances did you give in the last academic year?”

These data were gathered after receiving an affirmative collaboration response from four preuniversity music training centers. Our research team traveled to those institutions to gather the information *in situ*. We assured participants that their participation was voluntary and that anonymity would be maintained. Ethics clearance was granted by the University of Zaragoza, who authorized the research project as part of a funded UZ2016-SOC-06 research program.

### Procedure

The analysis techniques in this investigation apply structural equations: SEM analysis allows comparison of several models to establish explanatory paths among a series of different population groups. Thus, as explained by Byrne (2010), one uses a constrained model as a point of departure in which both population groups – in our case, males and females – are initially regarded as equivalent. The estimation method applied here is maximum likelihood estimation. To analyze the models’ fit, we applied the indices normally employed in these models as reported by AMOS: the χ^2^ index and the normalized χ^2^ index (χ^2^/*df*); root mean square error of approximation (RMSEA); comparative fit index (CFI) (Byrne, 2010). Comparisons among models were made by χ^2^/*df* and the Akaike information criterion (AIC) index (Byrne, 2010).

After having subjected the data to several analyses, an improved adjustment is obtained, revealing internal relations among significant variables, as well as differences between males and females.

## Results

An initial descriptive analysis (Table 1) reveals significant differences (*p* < 0.05) between boys and girls in all the variables postulated in this study, except for the one referring to the perception of social support on the part of the parents (*F*_1__–__357_ = 0.003; *p* = 0.959). Taking into account the low size effects reported by the η^2^-value, boys report higher levels of self-efficacy in performance, in social support from teachers, and in a greater number of solo performances achieved; they likewise report a greater level of anxiety related with early context, a lower level of social support coming from peers, and a lower level of helplessness and MPA. The η^2^-value indicates that the effect size in the differences among MPA levels is moderate.

**TABLE 1:** Descriptive and analysis of variance.

	**Boys (*n* = 161)**		**Girls (*n* = 198)**		***F***	**Sig.**	**η^2^**
	**Mean**	***SD***	**Mean**	***SD***			
Performance self – efficacy	52.384	8.410	50.285	9.277	4.935	0.027	0.014
Social support – parents	53.809	8.089	53.855	8.389	0.003	0.959	0.000
Social support – teachers	50.086	8.176	47.573	10.077	6.519	0.011	0.018
Social support – peers	14.633	5.289	16.408	5.199	10.197	0.002	0.028
Early context	10.801	2.841	10.086	2.444	6.570	0.011	0.018
Helplessness	29.199	8.796	31.364	8.366	5.677	0.018	0.016
MPA	32.950	11.806	39.535	14.118	22.326	0.000	0.059
Solo performances	2.077	1.054	1.820	0.9468	5.924	0.015	0.016

**FIGURE 2:**
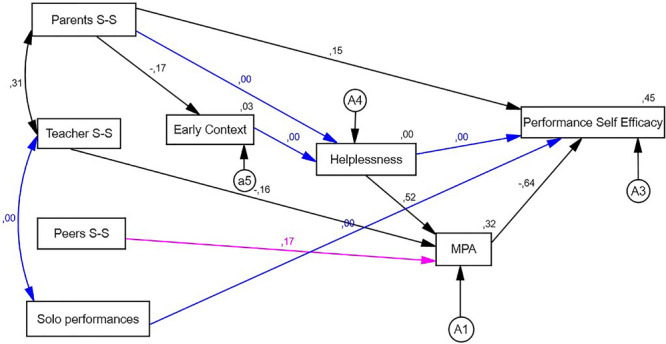
Final model: boys.

**FIGURE 3:**
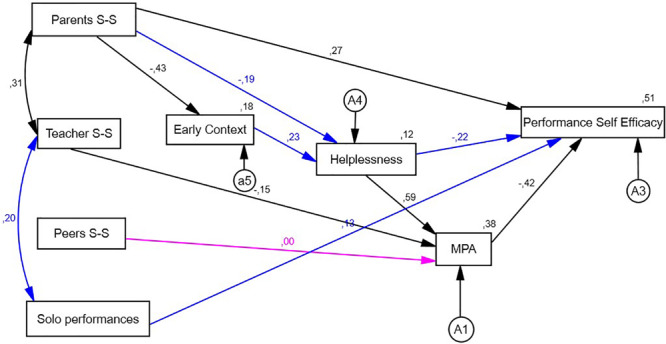
Final model: girls.

On the other hand, we find correlations between the explanatory variables of the model described in Figures 1–3. The correlations for boys (*n* = 161) show that there are only significant correlations between parent social support and teacher social support (*r* = 0.313; *p* < 0.001), whereas in the case of girls (*n* = 198), we find direct significant relationships between teacher social support (*r* = 0.331; *p* < 0.001), as well as between teacher social support and the number of solo performances given in a year (*r* = 0.231; *p* = 0.000) (Table 2).

**TABLE 2:** Correlations.

**Boys**	**Social support** – **parents**	**Social support** – **teachers**	**Social support** – **peers**	**Solo performances**	**MPA**	**Helplessness**	**Early context**
**Girls**							
Social support – parents	1	0.313**	0.097	0.075	−0.159*	–0.130	−0.170*
Social support – teachers	0.331**	1	0.138	0.085	−0.196*	–0.114	–0.072
Social support – peers	0.119	0.050	1	0.055	0.143	0.001	–0.103
Solo performances	0.102	0.231**	0.025	1	0.057	0.039	0.128
MPA	−0.272**	−0.264**	–0.035	−0.159*	1	0.535**	0.142
Helplessness	−0.283**	−0.206**	–0.077	–0.116	0.610**	1	0.094
Early context	−0.425**	–0.133	–0.012	–0.011	0.148*	0.307**	1

** The correlation is significant at the level 0.01 (two-tailed). * The correlation is significant at the level 0.05 (two-tailed).

On the basis of the theoretical explanation provided above, as well as of the first descriptive results and correlations, an initial model portrays the manner in which we sought to relate all variables in the same way in the case of males and females, furthermore assuming that the variables that evaluate early context, helplessness, MPA, and self-efficacy in musical performance could be the result of social support on the part of parents, professors, and peers and could likewise depend on the number of solo musical performances given in the course of the previous academic year (Figure 1).

The initial general model is equally applicable to males and females and has good fit with the data, thus: chi-square to df ratio (CMIN/*df*) = 1.913; CFI = 0.996; RMSEA = 0.051; AIC = 175.826. In both population groups, however, we observe a series of non-significant internal relations among variables (scaled to zero in Tables 3, 4).

**TABLE 3:** Standardized regression weights.

		**Boys**		**Girls**	
		**Estimate**	***p***	**Estimate**	***p***
Early context	Parents’ social support	0		−0.425	0.000
	Teacher social support	0		0	
	Peer social support	0		0	
Helplessness	Early context	0		0.228	0.002
	Peer social support	0		0	
	Solo performances	0		0	
	Teacher social support	0		0	
	Parents’ social support	0		−0.186	0.011
MPA	Helplessness	0.520	0.000	0.586	0.000
	Early context	0		0	
	Teacher social support	−0.160	0.014	−0.145	0.010
	Peer social support	0.165	0.011	0	
	Solo performances	0		0	
	Parents social support	0		0	
Performance self-efficacy	MPA	−0.643	0.000	−0.417	0.000
	Parents’ social support	0.153	0.009	0.268	0.000
	Early context	0		0	
	Solo performances	0		0.126	0.012
	Helplessness	0		−0.225	0.000
	Teacher social support	0		0	
	Peer social support	0		0	

**TABLE 4:** Correlations.

		**Boys**		**Girls**	
		**Estimate**	***p***	**Estimate**	***p***
Parents’ social support	Teacher social support	0.313	0.000	0.331	0.000
Parents’ social support	Peer social support	0		0	
Parents’ social support	Solo performances	0		0	
Teacher social support	Peer social support	0		0	
Teacher social support	Solo performances	0		0.313	0.000
Peer social support	Solo performances	0		0	

After having made the appropriate adjustments (scaling the regression weights and the non-significant correlations to zero, both in boys and in girls, and allowing for differences between the groups of girls and boys in the internal relations among variables), we obtained a model with better fit and parsimony than the initial one (CMIN/*df* = 1.021; *p* = 0.434; CFI = 0.998; RMSEA = 0.008; AIC = 138.784) (Tables 3, 4).

In boys as well as in girls, social support from parents exerted a direct significant relationship with autoefficacy for musical performance and with early context and perceived social support from teachers. Similarly, both the male and female categories feature a direct significant relationship between teacher social support and the specific MPA factor. Furthermore, in both groups, we noted that helplessness exerted a direct relationship with the specific MPA factor, and the latter had a direct relation with autoefficacy for musical performance (Tables 3, 4).

Comparing Figures 2, 3, we thus found common elements that help explain autoefficacy for music performance in both population groups. These explained 45% of that factor’s variance in the group of boys and 51% of variance in the group of girls.

We nevertheless noticed differences between the internal associations among variables. In the case of boys, there was no significant relation between parent support and the helplessness factor. Neither was there a relationship between early context and helplessness, nor was helplessness significantly related with self-efficacy for musical performance. Teacher support, as perceived by boys, had no significant relationship with the number of performances given in the past academic year. In contrast, all of these relations were significant in the case of girls.

In the case of girls, there was no significant relationship between peer support and the specific MPA factor, whereas that same relationship was significant for boys.

## Discussion and Conclusion

This study examined musical self-efficacy and its relationship with other sociodemographic and pedagogical variables, as well as with social support and MPA. Taking Bandura’s theory of self-efficacy as our point of reference, we postulated a model in which social support, individual performances, and dimensions of MPA according to Barlow’s model were posited as exogenous and mediating variables. Our ultimate objective was to better understand the emergence of musical self-efficacy as a means of improving musical training. Carried out with a sample of 359 Spanish students, our study validated the model, which accounts for approximately 45% of self-efficacy in males and 51% thereof in females on the basis of three social support components: parents, teachers, and peers, as well as a variable that measures the number of solo public performances already undertaken.

However, not all relationships postulated in the theoretical model turned out to be significant, neither were they equal in boys and girls. As mentioned in *Results*, males and females approach their belief in self-efficacy in a significantly different manner. Parental support exerts an influence on self-efficacy in musical performance both in boys and in girls; however, in the case of girls, it does so through the dimensions of early context and helplessness. Previous results in Spain have already revealed a greater influence of family relationships on the personal development of girls (Orejudo et al., 2012). Basing ourselves on the previously expounded explanation of the self-efficacy construct from the angle of social support, we see that parents play a fundamental role in fostering self-efficacy beliefs in males and in females. This is in agreement with other investigations, such as Ryan et al. (2000). Further studies reinforce this finding, highlighting a series of aspects generated by the parents in this context of interaction: goals, expectations, participation in music-oriented activities, feedback regarding self-esteem, and emotional support (Sichivitsa, 2007; McPherson, 2009; Nogaj and Ossowski, 2015). As stressed above, these are key factors in the development of musical self-efficacy.

Nevertheless, and somewhat contrarily to the findings of Raufelder et al. (2018), the teacher’s role is not at all clear as an ultimate explanation of music student self-efficacy. We observe that, in both males and females, the teacher’s role did not directly account for self-efficacy, but its influence thereupon was mediated by the specific anxiety cognitions. In the case of both male and female subjects, the role of their teachers was not significantly related with the cultivation of certain beliefs in autoefficacy in terms of performance, but instead exerted an influence on certain levels of MPA, which, in turn, triggered a differing perception of self-efficacy. Nevertheless, further research should aim to clarify the mechanisms of teacher influence. Thus, for instance, Nogaj and Ossowski (2015) point out that teachers, particularly teachers of a musical instrument, are an important source of evaluative information, which helps the student form their musical self-concept. We can interpret this result from the angle of the mediating relationship between self-concept and MPA.

Thus, a relationship between teachers and students can be established as a type of interaction in which three factors play a role: the emotional factor, the organizational factor, and pedagogical support (Pianta and Hamre, 2009). Along the same lines, Rimm-Kaufman et al. (2015) have suggested that the degree of engagement between students’ and their teacher is different in males and females throughout adolescence, perhaps because girls tend to commit more firmly to studying. And as emphasized by Engels et al. (2016), a positive relationship between the teacher and the student is associated with greater levels of engagement toward the learning task over time. That significant relationship between girls and their teachers helps to explain, at least partially, the reason for the differences between males and females that affect the significant relation between teacher support, the number of solo public performances, and the influence thereof on self-efficacy beliefs. In other words, because the relationship between teacher support and the number of performances is significant in the case of girls (possibly due to females’ greater commitment to the learning task and to teachers, as pointed out in Heyder and Kessels, 2015), it can be assumed that this relationship exerts an influence that is directly proportional to their autoefficacy beliefs.

The case is different for boys, for whom no significant relationship was found between teacher support and the number of performances, probably because boys display lower levels of engagement with their teachers and with the learning task, as suggested in the literature. Thus, in the case of boys, no significant relationship was found between the number of solo performances and their level of autoefficacy in musical performance.

Finally, there is little evidence regarding the manner in which peers can contribute to an individual’s musical progress. In our study, we have noted how a greater perception of peer support is not only related with differences between levels of helplessness in males and females, but in the case of males it is also directly proportional to the specific MPA level, whereas in females, it presents no significant relationship with that factor. On the other hand, our results do partially agree with those obtained by Schneider and Chesky (2011), for whom peer support displays no significant relation with the impact of anxiety on performance, or with physical or cognitive symptoms of MPA. In their study, however, peer support does display a significant relation with the degree in which students are capable of managing and controlling MPA. At any rate, the effect is not very large, thus bringing our results in line with previous studies. Hallam et al. (2016) found that peer support is much more related to social life and activities that revolve around music as a social activity, but does not provide an evaluative contribution to a student’s instrumental capacities. Nogaj and Ossowski (2015) find no relation between peer support and musical performance results; nevertheless, our results uphold the notion that, at least in the case of boys, peers can exert a certain influence as a source of support for controlling anxiety as related to public performances. Once more, gender differences stand out in this result, probably due to the finding that peer influence exerts a greater influence upon the development of boys than girls (Orejudo et al., 2012). Moreover, in the case of music students, a certain peer network could likewise be connected with music and with the development of musical identity, which plays a key role in the progress an individual can achieve in a musical career (Hallam et al., 2016). Thus, the type of social support evaluated in our scale – defense against bullying and mockery – would be qualitatively different from the type of support analyzed in other studies. Instead of indicating direct support for the exertion of musical activities, it could instead be pointing toward a negative conditioning effect in the face of criticism, which plays a key role in MPA.

Perhaps our most interesting finding consists in the observed differences in terms of gender related with the mediating function of helplessness and the role it plays in explaining perceptions of self-efficacy. Our results are certainly in line with previous studies that have opened up this line of research (Barber and Harmon, 2002; Filippello et al., 2015). It would seem that parental social support neither has a mediated relation with the helplessness factor, nor with early context, which would help explain self-efficacy perception in the case of males.

Following on, and in accord with Kenny and Holmes (2015) and Kenny et al. (2016), an explanatory relation seems to exist for the variance of the helplessness factor in females via direct action of parent support and relations with music study in an early context. This relationship, in turn, seems to have the capacity to explain females’ musical self-efficacy. It would, however, normally apply in boys as well, yet in our results it does not. In the case of males, it would seem that helplessness acts as an exogenous variable that does not display any relationship whatsoever with any of the three social support agents we evaluated (parents, teachers, and peers). Instead, it exerts a direct influence on the specific MPA factor, which, in turn, has an inversely proportional relationship with self-efficacy. At any rate, our results corroborate those of previous studies regarding the importance of helplessness in MPA (Kenny et al., 2004; Orejudo et al., 2017; Coşkun-Şentürk and Çırakoğlu, 2018). The only study that previously attempted to note differences between genders did not find any (Coşkun-Şentürk and Çırakoğlu, 2018); thus, this finding would be confirmed for both boys and girls.

Finally, in our model, we find another particularly relevant result: the influence of solo performances on musical self-efficacy. This result is of key importance within the framework of Bandura’s (1986) self-efficacy model, because it confirms the role played by mastery experiences. However, in our case, this result only appears significantly in the case of girls. Girls, moreover, were more likely to display a significant relationship between the number of solo performances and their teacher’s support. In these cases, we can assume that the opportunities for solo performance were mostly determined by the degree of commitment agreed upon between teacher and students. No other studies have analyzed this differentiated role exerted by the gender factor, although certain studies have tried to relate solo performance with the development of MPA (Casanova et al., 2018; Coşkun-Şentürk and Çırakoğlu, 2018). In the only study that carries out a comparison between genders, Coşkun-Şentürk and Çırakoğlu (2018) report a significant relationship between the two factors in both genders. Both in our case (β = -0.17) and in theirs (β_boys_ = 0.187; β_girls_ = 0.147), the regression weights are not particularly strong; thus, it might be possible that our study did not manage to detect those effects in the case of boys. We only took solo performances into account; other types of performances might nevertheless also exert an effect on self-efficacy.

Beyond these concrete results and their discussion, it is important to bear in mind that, according to Jinks and Lorsbach (2003), the relationship between self-efficacy and achievement is determined by the influence of self-efficacy on motivation and learning, on perseverance, on objectives, and on the use of cognitive, affective, metacognitive, and self-monitoring factors in performance (Schunk, 1991; Pajares, 1997). The optimum level for self-efficacy to work correctly in a given task is when the subject’s perceptions and opinions of their own abilities are slightly higher than their actual potential to adequately carry out the task (Pintrich and Schunk, 1996).

As stated previously, our perceptions of self-efficacy influence not only our achievement, but also the decisions we make with regard to certain actions, as well as our willingness to maintain them. Personal perceptions become much more complex when the action to be carried out includes some kind of performance in public – a typical situation within the artistic domain, where the purpose of a performance is to exercise one’s profession competence in front of an audience (artist CVs, for instance, tend to include a list of previous public performances).

According to self-determination theory (Ryan and Deci, 2017), all humans feel a need for the fulfillment of three basic psychological needs: competence, connectivity, and autonomy. These basic needs are essential conditions to the improvement of motivation and achievement within the context of various forms of social support.

Practical implications arising from our results thus suggest that specific needs should be addressed within the context of the Spanish education system. We wish to conclude by briefly describing several of them. It is urgent that initial or ongoing pedagogical training should enable teachers to understand the concrete implications of the above-expounded theory and results; such knowledge would enable them to adopt a fundamentally different approach. Thanks to such knowledge, they could become more aware of the different learning modes observed in human beings. We do not all acquire or organize knowledge in the same fashion (Nielsen, 1999; Torrado, 2003; Burwell, 2005; Bautista et al., 2006; Pozo et al., 2006; Ruiz et al., 2006; Kirschner and van Merriënboer, 2013). For instance, just by recognizing the difference between boys and girls in terms of knowledge acquisition (observed in this and other studies), teachers can start to modify their teaching–learning strategies.

Furthermore, it becomes indispensable for instrument teachers to fulfill the function of mentoring, counseling, and tutoring their students. Music teachers not only need to be excellent transmitters of knowledge and models of behavior, but they also should also act as sensitive counselors and rigorous guides in the study of a musical instrument (Lehmann and Ericsson, 1993, 1996). They should familiarize themselves more closely with each student’s character and personal history, in order to appreciate differences among individuals and the pedagogical implications those differences may have.

It is also necessary to establish true social support that motivates students to face the challenges involved in music studies while avoiding dropout. Such social support should be potentialized and multiplied via the collaboration of all agents with whom the student is involved. Professors can learn to better adapt academic study repertoire by selecting those works that, within the corresponding level, best adapt to the individual student’s personal characteristics (Jorgensen, 2000; Hallam, 2002; Juslin, 2003; Bernabé, 2013), while implementing innovative strategies and methodologies borrowed from other branches of pedagogy such as gamification, project workgroups, and the incorporation of certain new technologies in the practice routine. The student’s family should offer the same type of support within a culture that encourages teachers and families to “pull in the same direction,” thereby ensuring that the student is positively reinforced (an essential motivational factor).

In order to implement such changes, we suggest that the Spanish music teaching curriculum be fundamentally revised. A new syllabus design is needed that focuses on the development of musical as well as nonmusical skills and capabilities (Martín and Cervi, 2006; Vernia, 2016), instead of merely transmitting content or preparing students for concrete repertoire and/or for specific tests. In other words, we recommend a curriculum that stresses the importance for teachers to motivate, prepare, and engage their students to attain the desired level of artistic endeavor in an individually tailored way. Curricula would need to take each individual student’s needs into account, thereby preparing them for public performances in a way that would make them feel capable of rising to the challenge. Institutions of musical learning need to foster successive, gradually introduced activities that expose the students to mastery experiences through participating in performances in front of an audience from the first years of their musical training.

Among the skills and competencies one should acquire (Ericsson et al., 1993; Marchesi and Martín, 1998), there is one that is intimately related with autoefficacy: *learning to learn* (Williamon and Valentine, 2000; Williamon et al., 2002; Pennesi, 2012). Music students devote a great deal of time learning their instrument. It is therefore essential to foster all activities that help them to become increasingly familiar with efficient study methods and techniques of time management, in addition to improving their self-knowledge while learning to respect and value their own individual limits. Thus, the student can gain a greater amount of independence while developing a proactive spirit of initiative.

One of the specific pending needs is that of fostering a true pedagogical connection between teachers and students (Sloboda, 1985; Marchesi and Martín, 1998). From the outset, all students need to be aware of the objectives of the activities they will be carrying out. It is important to work on certain types of content or to adequately grasp all that is being learned while gaining a greater appreciation of process-based learning (the need to master all the preceding subject matter in order to proceed along the learning process in a gradual, stepwise fashion while always building on the basis of what has gone before), in other words, to collaboratively involve students in everything that impinges upon their learning process (Zaragozá, 2009). Such understanding, communication, and transmission of learning content will certainly result in a greater degree of autoefficacy in students of the future.

In the preceding paragraphs, we have presented a series of possible suggestions for improvement derived from the practical implications of the present study. All of these suggestions are fundamentally related with individualization in the teaching–learning process. Further research regarding the improvement of general pedagogical procedures will need to be carried out in order to more fully understand how musical training can profit from such changes.

In this sense, even though the study presented is novel within the Spanish musical research and training context due to the analysis techniques it applies and the sample size it handles, the quantitative approximation shown here, while being robust and defendable, should be complemented in the future with a general mapping endeavor that uses longitudinal quantitative study techniques, as well as qualitative techniques to collect the opinions and explanations of both students and teachers.

In this way, the Spanish system will be able to put into operation, as other overseas countries have, long-term training models both for students and teachers that are based on solid evidence that demonstrate how musicians can gain experience and mastery of tasks in a way that allows them to survive in a highly competitive and demanding industry.

## Data Availability Statement

The datasets generated for this study are available on request to the corresponding author.

## Ethics Statement

The studies involving human participants were reviewed and approved by the CEIC Aragón (CEICA). Written informed consent to participate in this study was provided by the participants’ legal guardian/next of kin. Written informed consent was obtained from the individual(s), and minor(s)’ legal guardian/next of kin, for the publication of any potentially identifiable images or data included in this article.

## Author Contributions

All authors listed have made a substantial, direct and intellectual contribution to the work, and approved it for publication.

## Conflict of Interest

The authors declare that the research was conducted in the absence of any commercial or financial relationships that could be construed as a potential conflict of interest.
